# Co-occurrence of *Lactobacillus* Species During Fermentation of African Indigenous Foods: Impact on Food Safety and Shelf-Life Extension

**DOI:** 10.3389/fmicb.2022.684730

**Published:** 2022-04-07

**Authors:** Adekemi Titilayo Adesulu-Dahunsi, Samuel Olatunde Dahunsi, Titilayo Adenike Ajayeoba

**Affiliations:** ^1^Food Science and Technology Programme, College of Agriculture, Engineering and Science, Bowen University, Iwo, Nigeria; ^2^Microbiology Programme, College of Agriculture, Engineering and Science, Bowen University, Iwo, Nigeria; ^3^Department of Microbiology, Adeleke University, Ede, Nigeria

**Keywords:** Africa, fermented foods, starter culture, food security, *Lactobacillus* species

## Abstract

The benefits derived from fermented foods and beverages have placed great value on their acceptability worldwide. Food fermentation technologies have been employed for thousands of years and are considered essential processes for the production and preservation of foods, with the critical roles played by the autochthonous fermenting food-grade microorganisms in ensuring food security and safety, increased shelf life, and enhanced livelihoods of many people in Africa, particularly the marginalized and vulnerable groups. Many indigenous fermented foods and beverages of Africa are of plant origin. In this review, the predominance, fermentative activities, and biopreservative role of *Lactobacillus* spp. during production of indigenous foods and beverages, the potential health benefit of probiotics, and the impact of these food-grade microorganisms on food safety and prolonged shelf life are discussed. During production of African indigenous foods (with emphasis on cereals and cassava-based food products), fermentation occurs in succession; the first group of microorganisms to colonize the fermenting substrates are lactic acid bacteria (LAB) with the diversity and dominance of *Lactobacillus* spp. The *Lactobacillus* spp. multiply rapidly in the fermentation matrix, by taking up nutrients from the surrounding environments, and cause rapid acidification in the fermenting system *via* the production of organic compounds that convert fermentable sugars into mainly lactic acid. Production of these compounds in food systems inhibits spoilage microorganisms, which has a direct effect on food quality and safety. The knowledge of microbial interaction and succession during food fermentation will assist the food industry in producing functional foods and beverages with improved nutritional profiling and technological attributes, as *Lactobacillus* strains isolated during fermentation of several African indigenous foods have demonstrated desirable characteristics that make them safe for use as probiotic microorganisms and even as a starter culture in small- and large-scale/industrial food production processes.

## Introduction

Fermented foods have been in existence since antiquity and are consumed by different regions globally because of the numerous nutritional values conferred on humans. For decades, the physiology and the functionalities of many important microorganisms associated with fermented foods have been studied, and this has assisted in the development of foods with improved flavor, taste, texture, and consistency (Marco et al., 2020). In Africa, fermented foods constitute the main dietary components, as many foods are fermented spontaneously before consumption with the predominance of lactic acid bacteria (LAB). These fermented foods and beverages serve as a vehicle for important microorganisms, thus playing essential roles in humans when consumed. Traditional fermentation of food is a natural method used in preserving different foods and beverages produced and thus extends the food shelf life, improves food palatability and food digestibility, and inhibits undesirable microorganisms, which results in improvement of food nutritional values. All these positive characteristics that impacted the fermented foods are dependent on the functional and technological roles displayed by autochthonous microorganisms present in the food substrates. Yeast and LAB are the groups of microorganisms that perform fermentative roles and flavor production during food fermentation, with LAB being the predominant microbes isolated from African indigenous fermented foods (cereals and tubers), although other microorganisms such as *Bacillus* spp., acetic acid bacteria, and molds have been reportedly isolated during production of some other fermented foods: legumes, wine, etc. (Adesulu-Dahunsi et al., 2017a, 2020; Chaves-López et al., 2020). Studies have shown that during fermentation of African fermented cereal-based and cassava-based foods, growth is firstly initiated by the LAB.

In Africa, production of several foods consumed by the populace relies on the fermentation processes; food security and shelf life are improved because of the biopreservative role played by the autochthonous microorganisms, which is advantageous to the community where there is poor access to electricity and refrigerating systems (Adesulu and Awojobi, 2014). Fermentation in food production is simply brought about by different activities of microorganisms and their enzymes to enhance the bio-accessibility and bioavailability of nutrients present in the raw food substrates, thus improving the organoleptic properties, improving digestibility, extending the food shelf life, inhibiting undesirable microorganisms, and serving as a vehicle for delivery of probiotics in humans (Diaz et al., 2019; Marco et al., 2020). Fermentation causes changes in both the physical and chemical properties of foods. It is described as the natural way of enhancing vitamin contents, essential amino acids, and sensorial properties of foods (Sharma et al., 2020). Food fermentation technologies are carried out at the household level *via* spontaneous fermentation consisting of mixed cultures of different species of autochthonous microbes, and back-slopping (re-inoculation of a previous successful fermentation batch into the new set to be fermented, which results in the dominance of best-adapted strains) has been carried out for several decades. In the last 2–3 decades, with increasing research and development, discoveries in the use of industrial-scale technology are preferred, i.e., inoculation of “defined starter culture” (pre-cultured single or mixed strains of microorganisms with functional and probiotics characteristics) in controlled fermentation processes; the latter is becoming a necessary under-regulated process to increase automated production, leading to improved and consistent fermented food quality for commercial purposes.

Several advantages of these fermented food products have been reviewed by various researchers (Melini et al., 2019; Şanlier et al., 2019; Sharma et al., 2020; Tsafrakidou et al., 2020), including the following:

• Production of safe food products.
• Reduction in cooking time.
• Production of foods with extended shelf life.
• Improvement of the organoleptic properties of fermented food products/enhancement of food sensorial characteristics.
• Increases mineral and trace element bioavailability in food/enrichment of food nutritional contents and digestibility.
• Elimination of cyanide compounds in cassava tubers and other harmful substances that may be present in cereals.
• Prevention of diseases or infections such as diarrhea and salmonellosis.

The population dynamics of microbes during fermentation using phenotypic and culture-dependent techniques have resulted in the understanding of different important roles that exist in the microbial consortia (Oguntoyinbo and Narbad, 2015; Adesulu-Dahunsi et al., 2017b). The invention of meta-omic methods (metagenomics, metatranscriptomics, metaproteomics, and metabolomics) has greatly complemented the culture-dependent techniques for strain-level characterization of food microbiota during fermentation processes and has also assisted in the studying of strains’ performances and microbial interactions of the important microorganisms within the food matrix and the systematic analysis of microbial metabolism and responses to the external/environmental factors (Liu et al., 2005; Makarova et al., 2006; Walsh et al., 2017; Taylor et al., 2020). The understanding of different roles displayed by food-grade microorganisms present in spontaneously fermented food products is crucial and has helped in the optimization of the final food quality, bringing about improved food safety. This review gives an overview of the impact of the activities and predominance of *Lactobacillus* microbiota during the production of African indigenous foods and the industrial importance of their interactions in cereal-based and cassava-based fermentation, their application in optimized or controlled food fermentation over mixed culture/uncontrolled fermentation, and the impact of *Lactobacillus* species co-occurrence in the fermenting food matrix on product development and safety.

## Predominance of *Lactobacillus* spp. During Fermentation of African Indigenous Foods

Lactic acid bacteria perform the function of converting carbohydrate in food substrates into organic acids (principally lactic acid) and can also produce a wide range of metabolites (Rodríguez et al., 2019). Lactic acid bacteria is a common name given to the family *Lactobacillaceae* (Pfeiler and Klaenhammer, 2007). These groups of microorganisms are Gram-positive, acid-tolerant, mainly lactic acid producers, non-sporulating groups of important bacteria widely reported to perform fermentative roles in several African fermented foods and beverages, and some genera, most importantly the genus *Lactobacillus*, have a “Generally Recognized As Safe” (GRAS) status; these characteristics displayed by LAB affect the storage quality, thereby extending the shelf life and safety of many fermented foods and beverages (Oguntoyinbo and Narbad, 2015; Adesulu-Dahunsi et al., 2018). The most common LAB genera frequently reported to be associated with African indigenous fermented foods are *Lactobacillus, Lactococcus, Leuconostoc, Pediococcus*, and *Weissella. Lactobacillus* constitute an important genus within the phylum Firmicutes, class Bacilli, order Lactobacillales, and family Lactobacillaceae. Recently, the International Scientific Association for Prebiotics and Probiotics (Marco et al., 2021) reported that the genus *Lactobacillus* comprises over 250 species; the species that are more closely related (i.e., those that share similar physiological traits) are grouped under the same genus, *Lactobacillus* spp., with desirable probiotic attributes, and are now renamed, including the following: *Lactobacillus casei* (renamed as *Lacticaseibacillus casei*), *Lactobacillus paracasei* (renamed as *Lacticaseibacillus paracasei*), *Lactobacillus rhamnosus* (renamed as *Lacticaseibacillus rhamnosus*), *Lactobacillus plantarum* (renamed as *Lactiplantibacillus plantarum*), *Lactobacillus brevis* (renamed as *Levilactobacillus brevis*), *Lactobacillus salivarius* (renamed as *Ligilactobacillus salivarius*), *Lactobacillus fermentum* (renamed as *Limosilactobacillus fermentum*), and *Lactobacillus reuteri* (renamed as *Limosilactobacillus reuteri*) (Marco et al., 2021). Zheng et al. (2020) also proposed the reclassification of the genus *Lactobacillus* into 25 genera; the classification includes the genus *Lactobacillus* (*Lactobacillus delbrueckii* group), *Paralactobacillus*, and 23 other novel genera. *Lactobacilli* are predominant organisms involved in the fermentation of cereal-based foods and beverages in Africa. During lactic acid fermentation, LAB at the initial stage of fermentation improve the flavor, shelf life, nutritional values, and digestibility of fermented foods. The predominance and the involvement of *Lactobacilli* especially [*L. plantarum* (*Lactiplantibacillus plantarum*) and *L. fermentum* (*Limosilactobacillus fermentum*)] during food fermentation have been reported by several researchers (Oguntoyinbo and Narbad, 2015; Adesulu-Dahunsi et al., 2017a; Naghmouchi et al., 2019).

*Lactobacillus* species are widely applied in food production and are known for their fermentative ability, health, and nutritional benefits. The role of LAB in the safety and quality of fermented foods around the globe has been reviewed by several researchers (Antara et al., 2019; Chaves-López et al., 2020; Goel et al., 2020). Strains of *Lactobacillus* play key roles during food fermentation by contributing immensely to the microbiological safety and extension of food shelf life. During fermentation, *Lactobacilli* usually outcompete other microorganisms through the production of organic acids, which are inhibitory to potential competitors, leading to the elimination of pathogenic microorganisms. The co-occurrence and activities of the LAB species in the fermenting food matrix and their functional and technological roles during food production have a major impact on the food safety, nutrition, sensory characteristics, and shelf life extension, as these LAB species have been found to inhibit pathogenic microorganisms that may be present in the fermentation through increased pH and production of lactic acid, and removal of toxic compounds (Behera et al., 2018; Douillard and de Vos, 2019; Virdis et al., 2021). In Africa, diverse foods are produced from plant sources and animal milk, and these foods are naturally fermented into different types of edible products. Some of these plant-based fermented foods have their sources from cereal (maize, sorghum, and millet), cassava (tubers), African oil palm, fruits, and leafy vegetables (African locust beans, melon, castor oil seed, sesame, cotton seeds, fluted pumpkin bean, ripe plantain, the fruit of sand apple, fresh leaves of *Cassia obtusifolia*, etc.).

Although other types of microorganisms may be involved during the production of foods and beverages, the predominance of *Lactobacilli* during indigenous food fermentation is commonly associated with cereal-based and cassava-based foods; these substrates are processed into different types of foods and beverages. Most of the fermented cereal-based foods are consumed as complementary infant food and as cereal beverages in adults, while the fermented cassava-based foods are consumed as main course meals. Different fermented foods from cereals and cassava tubers include *mawé* (Beninese fermented sour dough); *gowé* (Beninese malted and fermented cereal-based beverage); *tchoukoutou* (Beninese home-brewed beer from sorghum); *ben-saalga* (Burkinabé fermented cereal gruel); *dolo* (Burkinabé fermented sorghum beer); Bikédi (Congolese fermented cassava tubers); *poto poto* (Congolese fermented maize dough); *cingwada* (East African retted cassava); *kisk* (Egyptian fermented cereal mixture); Bikédi, *foufou*, *chikwangue, mbalampinda, tutu, tsaba, minsela*, and *tsiya borde* (Ethiopian fermented beverages)*; injera* (Ethiopian sour fermented flatbread); *kocho* (Ethiopian fermented bread); *koko* and *koko* sour water (Ghanaian fermented millet porridge and drink)*; agbelima* and *kenkey* (Ghanaian fermented cassava-based food); *kisra* dégué (Ivorian fermented cereal gruel); *wômi, baca*, and *doklu* (Ivorian fermented cereal-based foods); *attiéké, placali*, and *attoupkou* (Ivorian fermented cassava-based foods); *uji* (Kenyan millet-based fermented porridge); *ogi* (Nigerian fermented cereal gruel); *gari, fufu*, and *lafun* (Nigerian fermented retted cassava); *pito* and *kunun zaki* (Nigerian fermented cereal-based beverages); *ikigage* (Rwandan fermented sorghum beer); *hussuwa* (Sudanese fermented sorghum food); (Sudanese fermented sorghum bread); *mbege, togwa*, and *kivunde* (Tanzanian retted cassava); *komé* (Togolese fermented cereal-based foods); *koko* and *bushera* (Ugandan fermented cereal beverage); and *mahewu* (Zimbabwean fermented cereal beverages) (Aboua et al., 1989; Keleke, 1996; Olasupo et al., 1997; Mestres et al., 1999; Kimaryo et al., 2000; Blandino et al., 2003; Lei and Jakobsen, 2004; Abriouel et al., 2006; Sawadogo-Lingani et al., 2007; Ndiaye et al., 2008; Hama et al., 2009; Ray and Sivakumar, 2009; Mukisa et al., 2012; Owusu-Kwarteng et al., 2012; Greppi et al., 2013; Sanni and Adesulu, 2013; Soro-Yao et al., 2013; Obinna-Echem et al., 2014; Djeni et al., 2015; Adesulu-Dahunsi et al., 2017b; Aka-Gbezo et al., 2017). Several examples of these African indigenous fermented foods and beverages (AIFFBs) ranging from cereal-based to cassava-based foods and beverages and the predominant *Lactobacillus* spp. associated are shown in Table 1.

**TABLE 1:** Africa indigenous cereal-based and cassava-based fermented foods and beverages and the predominant *Lactobacillus* spp. associated.

Products	Country of production	Substrates	Form	Predominant *Lactobacillus* spp. associated with the fermented products
*Aklui*	Bénin	Maize	Porridge	*Lactobacillus* spp.
*Poto-poto*	Congo	Maize	Dough	*L. plantarum, L. paraplantarum, L fermentum, L. gasseri, L. delbrueckii, L. reuteri, L. casei, L. brevis*
*Mawè*	*Côte d’Ivoire, Togo*	Maize	Basis for preparation of many dishes	*L. fermentum, L. reuteri, L. brevis*
*Banku*	Ghana	Maize	Dough	*Lactobacillus* spp.
*Kenkey*	Ghana	Maize	Staple food/Weaning food	*L. fermentum, L. reuteri*
*Masa*	Nigeria	Maize	Snacks	*L. fermentum, L. plantarum*
*Ben-saalga*	Burkina-Faso	Millet	Porridge as weaning food	*L. fermentum, L. plantarum*
*Dégué*	Burkina-Faso	Millet	Porridge	*Lactobacillus* spp.
*Dagnan*	*Côte d’Ivoire*	Millet	Dough	*Lactobacillus* spp.
*Womi*	*Côte d’Ivoire*	Millet	Fried cake	*Lactobacillus* spp.
*Busa*	Egypt	Millet	Beverage	*L. helveticus, L. salivarius, L. casei, L. brevis, L. plantarum, L. buchneri*
*Fura*	Ghana, Nigeria	Millet	Weaning food/Beverage for adult	*Lactobacillus* spp.
*Dalaki*	Nigeria	Millet	Porridge	*Lactobacillus* spp.
*Eko*	Nigeria	Millet	Staple food	*Lactobacillus* spp.
*Cere*	Senegal	Millet	Used for preparation of many dishes	*L. plantarum*
*Bushera*	Uganda	Millet	Beverage	*L. plantarum, L. paracasei* ssp. *paracasei, L. fermentum, L. brevis, L. delbrueckii* ssp. *delbrueckii*
*Gowé*	Bénin	Sorghum	Beverage for adult	*L. fermentum*
*Tchoukoutou*	Bénin	Sorghum	Beer	*L. fermentum, L. divergens, L. fermentum, L. fructuvoans, L. casei, L. acidophilus*
*Burukutu*	Bénin, Ghana, Nigeria	Sorghum	Beer	*Lactobacillus* spp.
*Dolo, Pito*	Burkina Faso, Ghana, Nigeria	Sorghum	Beverage	*L. fermentum, Lactobacillus delbrueckii* ssp. *delbrueckii, Lact. delbrueckii* ssp. *bulgaricus*
*Tchapalo*	Côte d’Ivoire	Sorghum	Beer	*L. fermentum, L. cellobiosus, L. brevis, L. coprophilus, L. plantarum.*
*Kome*	Togo	Sorghum	Dough as staple	
*Baca, Coco-baca*	*Côte d’Ivoire*	Maize, Millet	Porridge as weaning food	*Lactobacillus* spp.
*Kunun-zaki*	Nigeria	Millet or Sorghum	Beverage for adult	*L. plantarum, L. fermentum*
*Koko*	Ghana	Maize, Sorghum, Millet	Porridge	*L. fermentum, L. reuteri*
*Koko sour water*	Ghana	Maize, Sorghum, Millet	Weaning food/Dough as porridge	*L. fermentum, L. salivarius*
*Ogi*	Ghana, Nigeria	Maize, Sorghum, Millet	Cereal gruel	*L. plantarum, L. fermentum, L. brevis*
*Agidi*	Ghana, Nigeria	Maize, Sorghum, Millet	Food staple	*Lactobacillus* spp.
*Akamu*	Nigeria	Maize, Sorghum, Millet	Cereal gruel as staple, Weaning food	*L. fermentum, L. plantarum, L. pentosus, L. cellobiosus, L. mesenteroides, L. acidophilus, L. delbrueckii, L. lactis, L. casei*
*Kaffir*	South Africa	Malt of Sorghum, Maize	Beer	*Lactobacillus* spp.
*Bounganda, Loutoko, Biyoki*	Congo	Maize, Cassava tuber	Aqueous mixture maize flour and cassava flour	Not studied
*Banku*	Ghana	Maize, or Maize and Cassava	Dough as staple	*Lactobacillus* spp.
*Ubuswage, Imikembe*	Burundi	Cassava	Food staple	*Lactobacillus* spp.
*Ikivunde*	Burundi, Rwanda	Cassava	Food staple	*L. plantarum, L. brevis, L. fermentum*
*Myondo and Bobolo*	Cameroon	Cassava	Food staple	*Lactobacillus* spp.
*Mangbele*	Central Africa	Cassava	Food staple	*Lactobacillus* spp.
*Meduame-Mbong, cossette*	Central Africa	Cassava	Food staple	*Lactobacillus* spp.
*Chikwangue*	Congo	Cassava	Food staple	*L. plantarum*
*Ntobambodi*	Congo	Cassava (leaves)	Food staple	*L. fermentum, L. plantarum, L. lactis diacetylactis*
*Bikédi*	Congo	Cassava tubers	Food staple	*L. delbrueckii, L. fermentum*
*Mbalampinda, Tutu*	Congo	Cassava tuber	Starched and gelled cassava flour	Not studied
*Kokondé, Kokonte, Crueira, Alebo*	Côte d’Ivoire,	Cassava	Snack	Not studied
*Placali*	Côte d’Ivoire,	Cassava	Staple	Not studied
*Attoupkou*	Côte d’Ivoire	Cassava	Starchy cake	Not studied
*Kokondé (Kokonte)*	Côte d’Ivoire	Cassava	Food staple	Not studied
*Agbelima*	Côte d’Ivoire, Ghana, Togo	Cassava	Basis for preparation of many dishes	*L. brevis, L. plantarum, L. salivarius, L. fermentum*
*Attiéké*	Côte d’Ivoire, Burkina Faso, Bénin, Togo, Mali, Senegal	Cassava	Food staple	*L. plantarum, L. fermentum, L. cellobiosus, L. brevis*
*Inyange, Ivunde, Mokopa*	East Africa	Cassava	Food staple	Not studied
*Mboung*	Gabon	Cassava	Food staple	Not studied
*Akeyke*	Ghana	Cassava	Food staple	*L. plantarum, L. salivarius, L. brevis, L. fermentum*
*Abacha*	Nigeria	Cassava	Snack	*Lactobacillus* spp.
*Lafun*	Nigeria	Cassava	Food staple	*L. fermentum, L. plantarum*
*Gari*	Nigeria, Central and East Africa countries	Cassava	Staple	*L. plantarum, L. fermentum, L. brevis, L. pentosus, L. acidophilus, Lactobacillus* spp.
*Kivunde*	Tanzania	Cassava	Food staple	*Lactobacillus* spp.
*Mokopa*	Uganda	Cassava	Food staple	*Lactobacillus* spp.

*Muyanja et al., 2003; Lei and Jakobsen, 2004; Yousif et al., 2005; Vieira-Dalodé et al., 2007; Assohoun et al., 2012; Oguntoyinbo and Narbad, 2012; Greppi et al., 2013; Sanni and Adesulu, 2013; Adesulu-Dahunsi et al., 2017a; Bationo et al., 2019; Pswarayi and Ganzle, 2019.*

Diversity of LAB intraspecies interaction was reported to be associated with Ghanaian spontaneously fermented millet porridge and drink (*koko* and *koko* sour water), with *L. fermentum* as the predominant organisms. Assohoun et al. (2012) revealed the succession and predominance of LAB species during spontaneous fermentation of maize for the production of *doklu* (Ivorian maize dough). It was reported that after 24 h of fermentation, the percentage occurrence of *L. plantarum* was 64%, which was the highest, followed by other LAB: *Weissella cibaria* (22%), *Pediococcus acidilactici* (7%), and *L. fermentum* (7%); at 48 h fermentation time, *L. plantarum* spp. still predominate, having 56% of occurrence, and at the end of fermentation, the percentage of occurrence of *L. fermentum* was 100%. The predominance of LAB species especially *L. plantarum* in Nigeria traditional fermented foods and beverages is well documented (Oguntoyinbo and Narbad, 2015); this *Lactobacillus* strain plays significant roles during fermentation, and has been selected as a potential probiotic and starter culture due to the different properties it displayed, such as good acidification, hydrogen peroxide production, and variation in carbohydrate fermentation patterns (Rozos et al., 2018). From our previous study, the intraspecies differentiation of *L. plantarum* strains isolated from indigenous fermented foods was carried out using different molecular techniques for the selection of strains that can be used to produce foods with desirable functional properties or as an adjunct and/or starter culture during food fermentation processes. It is well established that the predominance of *Lactobacillus* spp. and their enzyme production capacity in African fermented foods have assisted in the breaking down of molecules of high-molecular-weight organic acids and compounds, bacteriocins, and hydrogen peroxide (Mokoena et al., 2016).

Lactic acid bacteria have been reportedly isolated at different fermentation stages, and researchers have recommended that the quality of the final food products is dependent on the microbial diversity, dynamics, and frequency of occurrence of these LAB species. Oyedeji et al. (2013) isolated and characterized the predominant LAB species during spontaneous fermentation of Nigerian cassava-based and cereal-based foods, *fufu* and *ogi*; the dominance in the population of the *L. plantarum* strains was observed, and the use of these LAB strains as a starter culture for production of fermented foods was suggested. Karaca et al. (2017) reported the predominance of *Lactobacillus* spp. (*Lactobacillus gasseri, L. fermentum, L. brevis*, and *L. casei*) during fermentation of *Dégué* (fermented millet beverages popularly consumed in some parts of African countries, namely, Burkina Faso, Ivory Coast, Senegal, Mali, Guinea, and Benin Republic).

## Roles of *Lactobacillus* spp. in Food Fermentation Industry as Functional Starters or Co-Cultures

**FIGURE 1:**
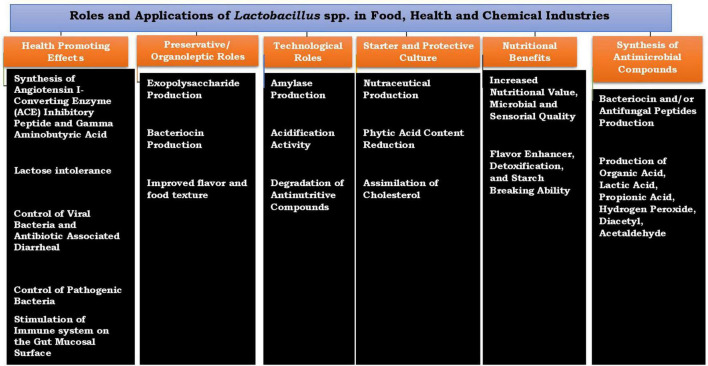
Roles and applications of *Lactobacillus* species in food, health and chemical industries.

In food processing industries, *Lactobacilli* constitute an important group of LAB, and the industrial importance of this *Lactobacillus* spp. cannot be overemphasized; they are commonly recommended as a starter culture for controlled fermentation of African indigenous fermented foods (Giraud and Cuny, 1997; Olasupo et al., 1997). The increase in demand for consistent and quality fermented products has resulted in the use of a starter culture for a more controlled fermentation process (Adebo et al., 2018; Masebe and Adebo, 2019). Controlled fermentation using a well-defined starter culture guarantees fast, consistent fermentation, the introduction of specific strains with exceptional functional properties, and food safety, though this technology is not widely practiced/used in many developing countries. *Lactobacillus* spp. associated with African indigenous fermented foods are known to exhibit many interesting beneficial traits and functional properties such as the production of the desired flavor, and antimicrobial production results in improved nutritional quality and safety of the food products and ultimately promotes the health status of the consumers. The roles and applications of *Lactobacillus* spp. are shown in Figure 1.

The use of a carefully selected functional starter culture with added nutritional benefits during microbial fermentation will enhance the production of fermented foods with improved quality (Nkhata et al., 2018). The technological effectiveness of LAB during food fermentation must be taken into consideration before selection as a functional starter culture or co-culture; some of these attributes include rapid acidification, development of antimicrobial compounds, probiotic features, and ability to improve the nutritional quality of fermented foods (Soro-Yao et al., 2014; Banwo et al., 2021). Many plant-based food substrates fermented with *lactobacilli* are reported to have high percentages of rhamnose (a naturally occurring deoxy sugar), and these sugars possess antioxidant properties that can be used as a substrate during *Lactobacillus* metabolism in cereal fermentation to produce vitamin-enriched foods and in combating nutritional deficiency in many African fermented cereal-based foods. During cereal-based fermentation, the dominance of *Lactobacilli* and other LAB with extracellular amylase production has resulted in the provision of metabolizable starch *via* hydrolysis and can be utilized for industrial fermentation processes, because of its acid production, which simultaneously improves the preservation and safety of fermented foods.

In cereal fermentation, the enzymatic activity of amylase displayed by *Lactobacillus* spp. in catalyzing the hydrolysis of amylose and amylopectin to yield fermentable maltose makes each strain desirable, important, and useful as a starter culture. In traditional food fermentation, using amylolytic lactic acid bacteria as a starter culture offers an advantage by combining both amylase production and acidification properties in a single strain. The raw starch binding ability of the α-amylase producing gene (*amyA*) of *L. plantarum* A6 (amylolytic strain isolated from retted cassava) with an unusual structure of the 3′ end of the α-amylase gene and the ability to break down raw starch has been successfully cloned and sequenced (Giraud et al., 1991; Giraud and Cuny, 1997). The *L. plantarum* A6 strain can synthesize large amounts of α-amylase that can be used as a starter culture during fermentation processes, resulting in the production of more lactic acid in the fermentation matrix.

Though beneficial starter cultures have not been fully utilized during the traditional fermentation of many cereal- and cassava-based products, fermented foods have probiotic potentials due to the *Lactobacillus* species present in the fermenting matrix. There are reports on the quality of some African fermented products that have been greatly improved upon using beneficial cultures (Nyanzi et al., 2013). *Ogi* was enhanced with a lactic acid starter culture to produce a functional food called *Dogik*. Nyanzi et al. (2013) reported the use of LAB associated with fermented foods to produce a starter culture possessing antimicrobial potentials; this starter culture has antimicrobial activities against diarrheagenic bacteria. During spontaneous fermentation of cereals to produce African fermented beverages, *akamu* and *ogi*, several LAB species such as *L. plantarum, Lactobacillus pentosus, Lactobacillus cellobiosus, Pediococcus pentosaceus*, and *Leuconostoc mesenteroides* were associated with *L. plantarum*, displaying the potential of being used as a starter culture (Nwachukwu et al., 2010). Oguntoyinbo and Narbad (2012) reported the multifunctional properties displayed by two strains of *L. plantarum* isolated from Nigerian cereal-based beverages (*L. plantarum* ULAG11 and *L. plantarum* ULAG24) and the potential of using these LAB strains for industrial processing of West African cereal-based foods and beverages, due to the probiotic function, amylase enzyme, and bacteriocin-producing ability exhibited by the strains (Mokoena et al., 2016).

## Biopreservative Role of *Lactobacillus* spp. in Fermented Food

The protective functions of many food-grade LAB in terms of improved food safety and food quality, against pathogenic and spoilage microorganisms during food fermentation, have attracted the attention of researchers. Biopreservatives are naturally occurring compounds that can be produced from microorganisms, plants, or animals. Some of the compounds produced by food-grade *Lactobacillus* spp. can be used to extend the food’s shelf life, because the antimicrobial metabolites released by these microorganisms may have some potential usage as natural preservatives that can be used to control or inactivate spoilage or pathogenic microorganisms in the fermenting food matrix; inhibition of the pathogenic or spoilage organisms that may be present in foods will result in increased food functionality and food quality (Pisoschi et al., 2018). Biopreservation using LAB and their metabolites connotes an alternative for improving food shelf life and safety (Reis et al., 2012). Many studies on the bacteriocinogenic effect of *Lactobacillus* spp. and its several applications in food preservations have been carried out. Many probiotic *lactobacilli* are reported to display inhibitory effects, which makes them useful as biological preservatives (Naghmouchi et al., 2019). The shelf life and safety of fermented foods can be enhanced through the biopreservative’s role as demonstrated by several LAB species in the form of competition to obtain nutrients during fermentation; this antagonistic form of interaction produces antimicrobial substances. Bacteriocin production by LAB is of great interest to the fermentation industry as some well-characterized bacteriocins have been reported to show a broad spectrum of activity on pathogenic and spoilage microorganisms (Cebrián et al., 2012; Naghmouchi et al., 2019; Vieco-Saiz et al., 2019).

Bacteriocins can be used as “biologically derived” preservatives; thus, food shelf life can be extended, and pathogenic organisms are inhibited without altering the product’s nutritional quality (Hugas et al., 2002; Ross et al., 2002). Lactic acid bacteria isolated from African fermented food are reported to demonstrate antimicrobial activity toward many pathogenic organisms, by the production of lactic acid and reduction of pH *in vivo* (Sanni et al., 2002). Many *Lactobacillus* species are known to be bacteriocin producers, and their presence in food inhibits the growth of pathogenic microorganisms. Olsen et al. (1995) reported the occurrence of antimicrobial compounds displayed by *Lactobacillus* species during fermentation of maize dough; the antagonistic interactions against pathogenic organisms in the fermented dough showed that the inhibitory compounds produced by the synergistic relationship between the strains of *L. plantarum* and *L. fermentum* were effective against both Gram-positive and Gram-negative bacteria, because of their ability to produce acids and bacteriocins. Adebayo et al. (2014) reported the use of bacteriogenic *Lactobacillus* strains (*Lactobacillus fermentum* and *Lactobacillus casei*) isolated from naturally fermented foods in Nigeria for effective control of spoilage and pathogenic microorganisms; the strains showed a broad range of activities and had a significant effect on the selected pathogenic microorganisms. Adedokun et al. (2016) demonstrated the use of a *Lactobacillus* strain as a food preservative agent. The antifungal activity of *L. fermentum* YML014 isolated from cassava-based fermented foods against food spoilage molds using tomato puree was investigated by Adedokun et al. (2016). It was found that the *Lactobacillus fermentum YML*104 showed strong antifungal activity against *Aspergillus niger, Aspergillus flavus*, and *Penicillium expansum*, and thus caused an increase in the shelf life of the tomato puree.

Production of antimicrobial compounds such as lactic acid, propionic acid, and diacetyl by LAB during fermentation results in a decrease in pH and inhibition of many pathogenic microorganisms. Rattanachaikunsopon and Phumkhachorn (2010) reported the inhibitory activity of reuterin produced by *Lactobacillus reuteri* against protozoa and fungi. Other inhibitory compounds such as organic acids, bacteriocins, and antifungal peptides produced by *L. plantarum* have been reportedly isolated from silage (De Vuyst and Leroy, 2007). Ben Omar et al. (2008) isolated 135 *Lactobacillus* species during the production of *poto poto* (Congolese fermented maize product) using species-specific PCR and 16S rRNA gene sequencing; 31 strains were identified as bacteriocin producers, and the bacteriocins produced by these LAB strains, *L. plantarum* (28) and *L. fermentum* (3), were identified as plantaricin and were found to display a broad spectrum of inhibition against the following pathogens: *Escherichia coli, Salmonella enterica, Enterobacter aerogenes, Bacillus cereus, Staphylococcus aureus, Listeria monocytogenes*, and *Enterococcus faecalis*. *Lactobacillus* spp. (*L. plantarum* F1 and *L. brevis* OG1) isolated from Nigerian fermented foods were reported to produce bacteriocins, which have a broad spectrum of inhibition against pathogenic and spoilage organisms; the *Lactobacillus* strains exhibited activities of 6,400 and 3,200 AU/ml, respectively, against *Escherichia coli* NCTC10418 and *Enterococcus faecalis* EF1, but did not inhibit *Candida albicans* ATCC10231 and *Klebsiella* sp. UCH15 (Ogunbanwo et al., 2003).

In another study, one *L. rhamnosus* and several *L. plantarum* strains isolated from *sha’a* (fermented maize beverage produced in Cameroon) produced bacteriocins that were able to inhibit both Gram-positive and Gram-negative bacteria, including species of the genera *Lactobacillus*, *Streptococcus*, *Bacillus*, *Salmonella*, *Shigella*, *Pseudomonas*, and *Klebsiella* and multidrug-resistant strains of the pathogens *E. coli* and *S. aureus* (Kaktcham et al., 1995). During food fermentations, *Lactobacillus* species have been reported to perform protective functions. Kaktcham et al. (1995) investigated the antimicrobial properties and protective functions of *Lactobacillus* spp. isolated during the fermentation of Cameroonian traditional cereal-based beverages and cow milk (*Sha’a* and *Kossam*). It was observed that 12 strains out of the 21 LAB species were reported to display inhibitory substances to proteolytic enzymes (Trypsin and Proteinase K) and the bacteriocins produced showed broad inhibitory activity against pathogenic microbes.

In cereal-based foods, LAB generally produces enzymes that assist in the breaking down of polysaccharides and other high-molecular-weight substances such as bacteriocins and hydrogen peroxide, thereby making it inhibitory for pathogenic organisms to survive (Mokoena et al., 2016). These LAB in fermented cereals also cause an increase in free amino acids and vitamin B groups, by breaking down antinutritional compounds, thus leading to the availability of iron, zinc, and calcium and production of gas and other volatile compounds and resulting in improved sensorial properties of the cereal-based foods and beverages (Blandino et al., 2003). Production of bacteriocins by LAB has also provided a good alternative to synthetic drugs and antibiotics. *Lactobacillus* produces antimicrobial ribosomally synthesized peptides, or “bacteriocins.” The LAB-producing bacteriocin has been reported to possess biopreservative characteristics, which makes them ideal food biopreservatives; some of these characteristics include the bacteriocin proteinaceous nature, *in vivo* non-toxicity and non-immunogenicity, inactivity against eukaryotic cells, thermoresistance against heat treatment, and broadened bactericidal activity (Naghmouchi et al., 2019; Vieco-Saiz et al., 2019). Several classes of LAB bacteriocins have their application in foods as food additives and control undesirable microorganisms in food (Heng et al., 2007). Nisin is the only FDA-approved bacteriocin produced from LAB species, and *Lactococcus lactis* is a widely studied biopreservative and has numerous applications as a food additive effective against foodborne pathogens and Gram-positive spoilage organisms during the production of cheese and dairy products. The effectiveness and application of bacteriocin in the food system have been widely reviewed (Cleveland et al., 2001; Naghmouchi et al., 2019). In the selection of bacteriocin-producing strains in food applications, the following must be put into consideration:

• The bacteriocin-producing strain should possess a “GRAS” status.
• The strain must display a broad spectrum of inhibition.
• The strain must be thermostable.
• The strain must possess beneficial effects and improve safety.

## Potential Benefits of *Lactobacillus* spp. as Probiotics

Probiotics are live microbes that impart health benefits to humans when an adequate amount is consumed (Hill et al., 2014). The significant effect of *Lactobacillus* as probiotic strains was first established at the beginning of the 20th century (1908) by the Russian scientist and Nobel Prize winner Elie Metchnikoff while working in Bulgaria; he hypothesized that the longevity of the Bulgarian centenarians was a result of the improved health benefits conferred upon daily intake of fermented yogurt containing *Lactobacillus bulgaricus* (Metchnikoff, 1908). Several indigenous fermented foods and dairy products have been studied to contain live microorganisms with potential health benefits (Lei and Jakobsen, 2004; Salmerón et al., 2015). *Obiolor* and *kunu-zaki* are examples of African fermented beverages with probiotic potentials and have indirectly impacted beneficial effects on the host; several attempts have been made to introduce probiotic drinks and beverages containing *L. rhamnosus* GR-1 and *Streptococcus thermophilus* in the form of a starter culture in the production of yogurt in Uganda, Tanzania, and Kenya (Salmerón et al., 2015; Nath et al., 2016; Westerik et al., 2020). The importance of probiotic microorganisms is so enormous to the development of the gastrointestinal tract (GIT), as probiotics have been described as a live microbial feed supplement that beneficially affects the host *via* improving the intestinal microbial balance (Steinkraus, 1995; Rezac et al., 2018). *Lactobacillus* has been well researched and is reported to be the most common microorganism associated with fermented foods, and most strains of this LAB species can reasonably confer a health benefit when an adequate amount is ingested (Hill et al., 2014; Sanders et al., 2018).

*Lactobacilli* have been reported as potential probiotics or employed as a starter culture, because of the essential roles they played during food fermentation. Up to 20 LAB species, consisting mainly of the genus *Lactobacillus*, were recognized by Canadian Food Inspection Agency [CFIA] (2013). Health Claims. Probiotic Claims. Summary table of acceptable non-strain specific claims for probiotics and eligible species for the claims. in Section 8.7.3 (Canadian Food Inspection Agency [CFIA], 2013). Many African fermented foods have been reported to confer probiotic attributes and enormous health benefits when consumed. These foods have probiotic potentials as so many isolated and characterized LAB are capable of surviving at low pH (2.5) and tolerating 0.3% bile salt for several hours. Application of viable cultures in controlling diarrhea among infants and their use as functional foods have been reported. Lei and Jakobsen (2004) reported the ameliorative effect in consuming *koko* sour water (Ghanaian spontaneously fermented millet drink) among diarrhea patients (diarrhea is the main cause of infant morbidity and mortality in developing countries). The *koko* and *koko* sour water millet porridge and drink were reported to contain an abundance of viable LAB, which are responsible for the beneficial effect conferred when these beverages are consumed. A similar report was obtained among Tanzanian children that were fed with *togwa* (lactic acid fermented cereal gruel) (Svanberg et al., 1992). A large number of LAB strains of the genus *Lactobacillus* and *Bifidobacterium* from fecal or vaginal sources are frequently reported compared to LAB of food origin, as many food-origin LAB are likely to be recovered from fecal sources.

*Lactobacillus* have been reported to have the ability to adhere to intestinal cells *via* coaggregation to form normal balanced flora and also produce compounds such as organic acids, hydrogen peroxides, and bacteriocins that are inhibitory to pathogenic organisms (Adeyemo et al., 2018; Ajayeoba and Ijabadeniyi, 2019). Several benefits are associated with the consumption of foods containing sufficient amounts of probiotic-containing well-defined and viable food-grade microorganisms (Dicks and Botes, 2010; Yadav et al., 2011; Furtado-Martins et al., 2013; Pasolli et al., 2020); these benefits include the following:

• Regulation of gut microbiota, which, in turn, leads to the improvement of the intestinal health of the host.
• Immune system development and prevention of infectious diseases.
• Improved nutritional quality of fermented products.
• Enhancement of the bioavailability of nutrients.
• Alleviation of allergies and of symptoms that may be associated with the lactose-intolerant individuals.
• Development of nutraceutical and/or functional foods.
• Production of bioactive compounds that enhances the functionality of foods and beverages.
• Lowering of serum cholesterol levels.
• Reduction in the risk of contracting some diseases.
• Prevention of cancer.

To harness the full LAB potentials of food origin, daily consumption of fermented foods is recommended. In the consensus statement on fermented foods issued by the International Scientific Association for Probiotics and Prebiotics (Marco et al., 2021), the panelists recommended that the term “Probiotic” should be used when demonstrated health benefits are conferred on humans by well-defined and characterized live and active microbes. From the conclusion, it is not enough that fermented foods contain viable and active microbes, but foods that will be labeled as probiotics must possess additional health benefits with proven safety and should ensure that such strain-specific products must be able to confer the required benefits on the host when the adequate amount is consumed.

## Fermented Foods and Human Gut Health

The inclusion and activities of bacteria as part of the human gut microbiota play valuable roles in the general health and wellbeing of man. The consumption of fermented foods is very crucial to gut health, as the LAB associated with these foods assist in boosting the good/beneficial microflora in the intestinal tract, resulting in the increased gut microbiome in the digestive system through which the human immune system is enhanced. Several investigators have recommended the consumption of traditional fermented foods as a veritable source of probiotics in humans (Mahasneh and Abbas, 2010; Jafari et al., 2011). Daily consumption of fermented foods containing live microorganisms will aid the delivery of substantial beneficial microorganisms to the GITs, as the presence of microorganisms associated with fermented foods in the GIT is sparsely documented (Zhang et al., 2016; Rezac et al., 2018). Some probiotic LAB isolated from the human gut have displayed their functionality in health improvement and have also been found to display some characteristics such as immune system improvement, inhibition of pathogenic bacteria, and modulation of epithelial cell permeability, which is relevant to therapeutic and/or prophylactic treatments against various diseases (Anderson et al., 2010; Ashida et al., 2011; Diaz Heijtz et al., 2011; Ahern et al., 2014; Wang et al., 2018).

Investigations have shown that fermented foods are the source of LAB represented in the gut microbiome. The predominance of these LAB in the human gut is a result of different contributing factors, age, lifestyle, geographical location, diet, and use of antibiotics, as the gut microbial composition is directly influenced by main dietary composition (Brewster et al., 2019). Several LAB species associated with fermented foods have been reported to have similar physiological traits with the strains known for improving gut health. The increasing interest in the use of probiotic LAB as a vehicle for drug delivery and treatment of GIT diseases has also been well documented (Mokoena et al., 2016; Wang et al., 2018). The probiotic microbes associated with fermented foods have immensely contributed to the protective roles in the gut as they are found to exhibit a strong inhibitory effect *in vitro via* organic acid production. *Lactobacillus* spp. also contribute to the healthy microbiota of human mucosal surfaces.

Gut bacteria perform several functions in the maintenance of human health, including production of vitamins, regulation of gut motility and development, maintenance of epithelial integrity by regulating tight junction permeability, inhibition of pathogenic microorganisms, and development of the central nervous system (CNS; Ashida et al., 2011; Erny et al., 2015; Mangalam et al., 2017). It has been proposed that gut bacteria are required to maintain epithelial integrity by regulating tight junction permeability. Karczewski et al. (2010) reported the ability of the LAB strain *L. plantarum* WCFS1 in enhancing the intestinal barrier of the host with intestinal disorders; the experiment showed the ability of the *L. plantarum* strain to regulate tight-junction proteins and to protect against chemical-induced disruption of the epithelial barrier. *L. rhamnosus* species have also been reported to demonstrate adherence to the gut epithelial tissue, resulting in the colonization of human GIT (Collins et al., 1998; Dunne et al., 2001).

Factors such as excessive antibiotic usage, stress, and diseases can alter the gut microbiota and may lead to dysbacteriosis. Consumption of fermented foods has been proven to be the easiest way of introducing potentially beneficial microorganisms to the GIT, though persistency of the probiotic LAB of food origin in the gut may not occur for a long time; it is recommended that regular consumption of these fermented foods will enhance the ability of the food-associated microorganisms to confer probiotic potentials in human (Derrien and Vlieg, 2015; Zhang et al., 2016). Thus, daily consumption of fermented foods is recommended, as it will enhance a healthy gut because of the innate beneficial microorganisms present in these foods.

## Conclusion

In Africa, the biotechnological application, development of bioreactor technology, and the use of a starter culture for food fermentation processes should be prioritized through technical skills training, provision of infrastructures, and government willingness in funding research in the area of upgrading fermentation processes and fortification of these foods with bio-enriched vitamins. Also, synergism among research institutes, universities, and manufacturing sectors will play a major role in bringing starter culture development and usage to the limelight in the food industry; this will guarantee consistent and safe food with improved shelf life. Several foods produced in Africa are fermented before consumption, and it is highly recommended that among the household food producers, good manufacturing practices should be effectuated from the point of collection of the raw materials/substrates because of the risks that are associated with improperly/unhygienically produced foods during the production of the final food products. The use of safe water should also be emphasized throughout the production/fermentation process; food should not be manufactured under poor hygienic conditions, and all these can be achieved by properly educating the food producers. The activities of fermentative LAB and most especially *Lactobacillus* strains have played substantial roles in ensuring food security and extension of food shelf life. Production of antimicrobial substances (most especially bacteriocins) by *Lactobacillus* spp. and the spectrum of antibacterial activity displayed by these species can find their wide applications in the food industry. As applications of *Lactobacillus* are enormous and wide usage as a probiotic and/or as a starter culture due to their ability to improve the product’s nutritional and technological features cannot be neglected, this LAB group occupies a central role during the fermentation process and significantly possesses many benefits as discussed in this review, with long and safe usage in the food industry. *Lactobacillus* species (those associated with African fermented foods) are described as key players during fermentation technology and have been reportedly involved in the enhancement of flavor, texture, and improved rheology with health-promoting characteristics, which can be applied in the production of functional foods, thus conferring beneficial attributes and consistency in food quality and thereby making the African food market/industry attractive. However, the use of appropriate metagenomic tools that will provide insights into the gene, structure, and functions of microbial strains with multi-functional properties during controlled fermentation will guarantee product quality and consistency, which will be accessible to all. In developing products with improved quality and safety, the inclusion of technologically relevant microorganisms (such as *Lactobacillus* strains) is crucial and will serve as sustainable interventions for the development of African-specific starter cultures. Development of starter culture technology for both small-scale and industrial-scale food production will lead to products of greater consistency, safe quality, and global acceptance, and all these should be the focus of African researchers and scientists. It can be observed that despite the rich diversity of food-grade microorganisms present in many African indigenous fermented foods, most of the well-defined starter cultures available in the shops and markets are not manufactured in Africa; therefore, there is a need to produce well-defined and well-characterized microbial cultures from autochthonous microorganisms peculiar to African foods.

## Author Contributions

AA-D: conceptualization and writing—original draft preparation. TA: writing part of the primary draft. AA-D and SD: technical editing of the manuscript and reviewing the manuscript for submission. All authors have read and agreed to the published version of the manuscript.

## Conflict of Interest

The authors declare that the research was conducted in the absence of any commercial or financial relationships that could be construed as a potential conflict of interest.

## Publisher’s Note

All claims expressed in this article are solely those of the authors and do not necessarily represent those of their affiliated organizations, or those of the publisher, the editors and the reviewers. Any product that may be evaluated in this article, or claim that may be made by its manufacturer, is not guaranteed or endorsed by the publisher.
